# A fungal exudate-inspired substrate mixture elicits an emergent growth phenotype and metabolic responses in *Pseudomonas putida*

**DOI:** 10.1128/msystems.00760-26

**Published:** 2026-07-24

**Authors:** Natalie Sadler, Elise Van Fossen, Grace Black, Vanessa Paurus, Nathalie Munoz, Yuqian Gao, Isaac Kwame Attah, Damon Leach, Joonhoon Kim, Sneha Couvillion

**Affiliations:** 1 Integrated Discovery Sciences Directorate, Pacific Northwest National Laboratory 497252, Richland, Washington, USA; 2 Integrated Discovery Sciences Directorate, Pacific Northwest National Laboratory, Sequim, Washington, USA; 3 Energy and Environment Directorate, Pacific Northwest National Laboratory 6865, Richland, Washington, USA; CNRS Delegation Bretagne et Pays de Loire, Nantes, France

**Keywords:** mixed substrates, predice phenomics, metabolic modeling, exudates, emergent growth phenotype, *Pseudomonas putida*

## Abstract

Microbes in natural environments often encounter diverse mixtures of organic compounds, yet how mixed substrate environments and their molecular composition shape microbial phenotypes remains understudied. Here, we examined how a defined fungal exudate mimic (FEM) mixture influences growth and metabolism in *Pseudomonas putida* KT2440 compared to individual substrates matched for total carbon and nitrogen. Growth on FEM initiated 2 h earlier than growth on glucose alone and exhibited both the lowest lag and shortest time to maximum biomass compared to individual substrates. Fructose was the only individual substrate that supported significantly higher maximum biomass than FEM, but exhibited a nearly 15-fold longer lag phase. Gas chromatography mass spectrometry analysis revealed dynamic temporal patterns of substrate utilization within the FEM mixture, with early preferential utilization of malate, followed by overlapping utilization of multiple substrates between 3 and 8 h. By integrating growth and substrate uptake kinetics with genome-scale metabolic modeling and validating model-predicted pathway activity using temporal proteomics, we show that experimentally constrained model predictions accurately captured substrate utilization dynamics across multiple FEM concentrations, and predicted temporal shifts in the dominant substrates supporting growth. Through this integrative experimental-modeling approach, we demonstrate that the mixed substrate FEM environment elicits an emergent growth phenotype characterized by the lowest lag, shortest time to maximum biomass, and relatively high maximum biomass in *Pseudomonas putida*, a combination of traits not simultaneously reproduced by any individual substrate.

**IMPORTANCE:**

How mixed-nutrient substrate environments influence bacterial growth and metabolism is not well understood and is challenging to predictively model. Using *Pseudomonas putida* KT2440, we show that growth on a defined mixture of substrates inspired by mycorrhizal fungal hyphal exudates produces a distinct and emergent growth phenotype characterized by a lower lag, shorter time to maximum biomass, and relatively high biomass accumulation when compared to individual substrates alone. This combination of growth traits is not simultaneously reproduced by any individual substrate, even when total carbon and nitrogen are matched. By integrating growth measurements and temporal substrate uptake data with dynamic flux balance analysis and proteomics, we demonstrate that metabolic responses to mixed substrates can be quantitatively interpreted. These findings highlight the importance of studying microbes under chemically realistic conditions and suggest that learning from naturally occurring molecular environments may provide new strategies for engineering microbial growth conditions and improving bioprocess performance.

## OBSERVATION

Understanding how bacteria adjust growth and metabolism in response to their molecular environment is a fundamental question in microbial ecology and biotechnology. In soil ecosystems, mycorrhizal fungal hyphae release mixtures of exudate compounds (e.g., organic acids, sugars, and amino acids) that are utilized by bacteria and help shape distinct hyphosphere microbial communities (1, 2). Phosphate-solubilizing rhizosphere bacteria, such as *Pseudomonas putida*, colonize the hyphae of arbuscular mycorrhizal fungi (3) and contribute to nutrient mobilization (4). Although fungal exudates are known to influence bacterial growth (5, 6), it remains unclear how such mixed substrate environments impact growth dynamics and metabolism compared to a single carbon source, even in well-characterized organisms such as *P. putida*. Although diauxie (sequential utilization) and co-utilization of multiple substrates have been previously described in complex media (7) and multi-substrate environments (8), it is unclear whether ecologically relevant, exudate-inspired mixtures produce unique growth phenotypes not reproduced by individual substrates.

**Fig 1:**
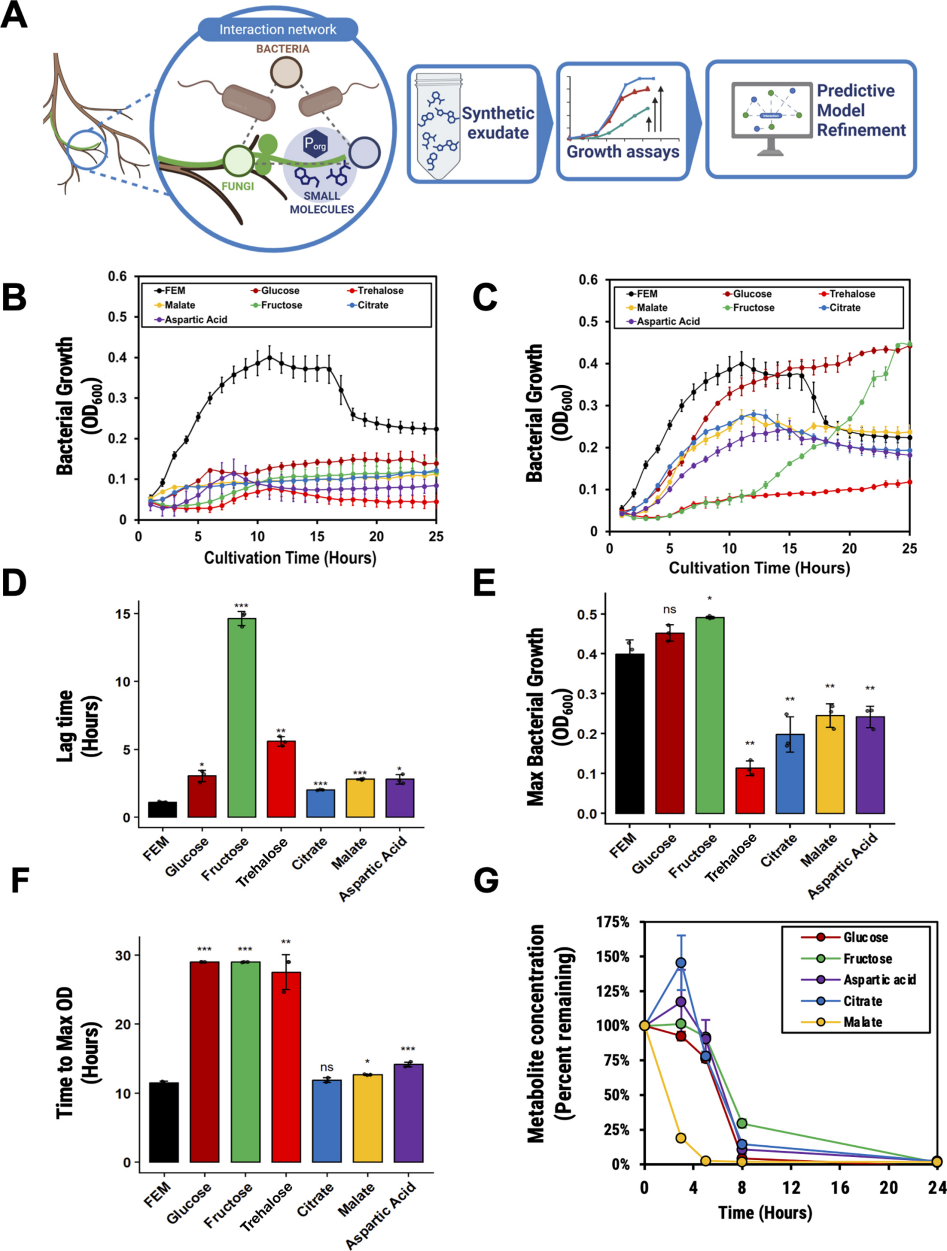
(**A**) Schematic overview of the experimental design. The metabolism of environmentally relevant small molecules by *Pseudomonas putida* KT2440 was characterized using time-resolved measurements of growth, substrate consumption, and proteomic profiles. These data were used to parameterize and refine predictive metabolic models. (**B**) Growth curves (OD_600_) of *P. putida* cultured with the fungal exudate mimic (FEM) or individual FEM substrates at concentrations equivalent to those in FEM. (**C**) Growth curves of *P. putida* cultured with FEM or individual substrates adjusted to match the total carbon and nitrogen content of FEM. (**D**) Lag times calculated from the growth curves shown in panel **C**. (**E**) The maximum growth, defined as the highest OD_600_ reached in panel **C**. (**F**) The time to max biomass, defined by the time required for each culture shown in panel C to reach its maximum OD_600_. (**G**) Time-resolved substrate concentrations in FEM cultures measured by GC-MS analysis of extracellular supernatants and presented as a percentage of remaining metabolite relative to the initial metabolite mass. Data in panels **B–G** are shown as mean ± SD (*n* = 5), and panel **G** as mean ± SD (*n* = 3). Statistical significance in panels **D–F** was determined using unpaired two-tailed Welch’s *t*-tests comparing each individual substrate condition to FEM. Asterisks indicate significance thresholds: ***, *P* < 0.001; **, *P* < 0.01; *, *P* < 0.05; ns, not significant.

To address this, we compared the growth dynamics of *P. putida* KT2440 on a defined fungal exudate mimic (FEM) mixture to growth on each individual substrate. We developed a predictive model combining Michaelis-Menten kinetics for modeling substrate uptake and genome-scale metabolic modeling for simulating cell growth. Kinetic parameters were estimated to match the substrate utilization in experimental data. This model was then validated against proteomics data to predict flux distribution and pathway activation under FEM (Fig. 1A).

The FEM was formulated based on published metabolomics profiles of fungal hyphal exudates (9, 10) from which we selected representative sugars, an amino acid, and organic acids that occur at high abundances (see Supplemental Information). The mixture contained glucose, fructose, trehalose, citrate, and malate (each at 100 µg C · mL^−1^) and aspartic acid (at 300 µg C · mL^−1^), with phytate included at 300 µM as a representative organic phosphorus source (6).

We compared growth kinetics for *P. putida* cultured with FEM and with the individual substrates either at the same substrate concentrations found in FEM (Fig. 1B) or adjusted to match the equivalent total carbon and nitrogen concentrations as FEM (Fig. 1C). In both cases, none of the single substrates reproduced the accelerated growth initiation observed with FEM. Lag phases for individual substrates (adjusted to match total C and N) were at least two times longer than the 1-h and 7-min lag observed for FEM cultures (Fig. 1D).

Maximum growth, defined as the highest OD_600_ reached in Fig. 1C, differed across substrates, and fructose was the only substrate that resulted in significantly higher cell density than FEM (Fig. 1E). Notably, fructose also exhibited both the longest lag phase, nearly 15× longer than FEM lag time, and the slowest progression to reach maximum growth. This likely reflects the requirement for induction of fructose-specific uptake and regulatory pathways in *P. putida*. (11) Consistent with the reduced lag, FEM cultures also reached maximum biomass significantly earlier than most individual substrates, except for citrate, where the difference was non-significant (Fig. 1F). This indicates that the FEM substrate mixture elicits an emergent growth phenotype arising from a combination of growth traits that are not simultaneously achieved under any individual substrate. These growth traits may provide an ecological advantage in the hyphosphere, where rapid resource acquisition and establishment of growth are beneficial.

To investigate the mechanisms driving these growth traits, we performed time-resolved GC-MS analysis of spent FEM medium to quantify substrate utilization over time (Fig. 1G; Table S1) and generate quantitative constraints for metabolic modeling. Distinct temporal patterns of substrate utilization were observed. Malate was rapidly consumed during early growth, with greater than 80% consumed by 3 h, and 98% consumed by 5 h. Between 3 and 8 h, glucose, citrate, aspartic acid, and fructose showed overlapping depletion, consistent with concurrent utilization of multiple substrates during this period of exponential growth. By 8 h, 96% of glucose, 89% of aspartic acid, and 86% of citrate were consumed. Fructose was the most abundant substrate remaining, with 71% consumed by this time point. By 24 h, all substrates were nearly completely depleted. Our results indicate early preferential utilization of malate following overlapping co-utilization of glucose, citrate, and aspartic acid during exponential growth, rather than the strict sequential consumption or catabolite repression that has been reported in the presence of certain substrates like succinate and lactate, to regulate carbon metabolism in *P. putida* (12). These findings suggest that substrate utilization dynamics in *P. putida* are strongly shaped by the specific composition of the surrounding molecular environment. Consistent with this interpretation, previous studies have reported different patterns of substrate uptake and metabolic reconfiguration when *P. putida* is grown in alternate mixed substrate environments, including complex media such as lysogeny broth (LB) (7).

**Fig 2:**
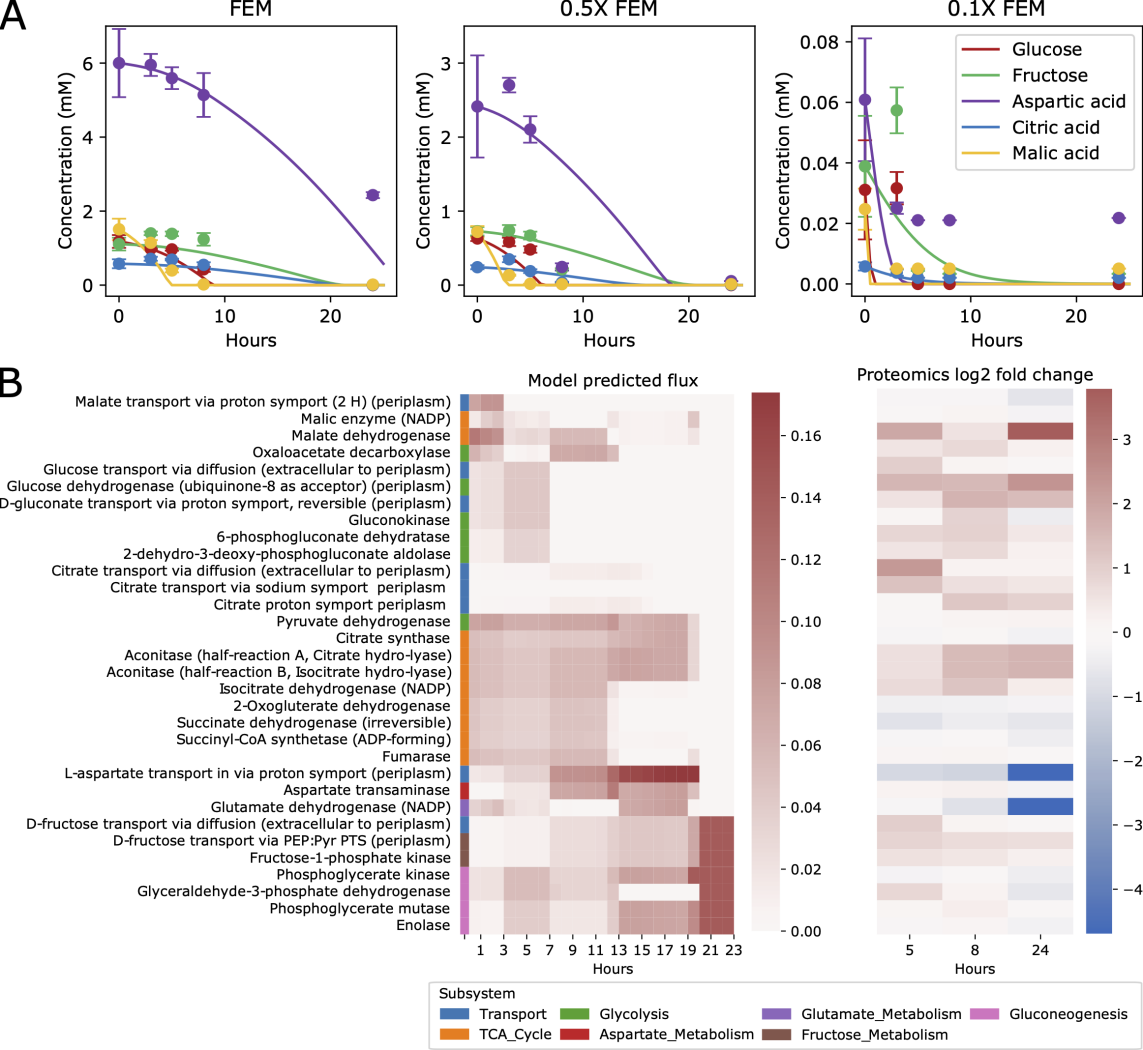
Metabolic modeling and proteomic analysis of *P. putida* metabolism in the mixed substrate FEM environment. (**A**) Experimentally observed (circles) and model-predicted (lines) substrate utilization in different levels of FEM. Experimental data are shown as mean ± SD (*n* = 3). (**B**) Model-predicted intracellular flux profiles over time compared to log_2_ fold changes from proteomics data. Metabolic reactions and enzymes involved in the top 24 reactions with the highest carbon flux and FEM substrate transport reactions are shown.

To determine whether our experimentally measured substrate uptake dynamics could explain the distinct growth phenotype observed under FEM conditions, we applied dynamic flux balance analysis (13) using the genome-scale metabolic model of *P. putida* iJN1462 (14). Dynamic flux balance analysis can predict microbial metabolism over time by coupling intracellular metabolic flux to external substrate changes, and has been used to study metabolic shifts during growth on multiple carbon sources (15). Substrate uptake was parameterized using Michaelis-Menten kinetics, and we estimated the model parameter values by fitting the model with time-course GC-MS data and growth data. Parameter fitting was performed on individual substrate kinetics using time-resolved substrate depletion and growth data obtained from FEM cultures across three FEM concentrations (1×, 0.5×, and 0.1×). Model predictions closely matched experimental substrate utilization dynamics across FEM concentrations from 1× to 0.1× (Fig. 2A; correlation coefficient = 0.986; *R*^2^ = 0.968), demonstrating strong agreement between simulations and experiments. This agreement across multiple FEM concentrations indicates that experimentally constrained modeling can robustly capture substrate utilization dynamics across varying FEM concentrations. Estimated uptake parameters and associated standard errors are reported in Table S2.

The model-predicted dynamic shifts in substrate utilization during growth on FEM. During the first 3 h, the major carbon flux supporting the growth was from malate (56% of the total carbon uptake), followed by glucose (23%). Aspartic acid, fructose, and citrate were 12%, 5%, and 3%, respectively. By 5 h, glucose (53%) became the dominant carbon contributor, followed by aspartate (28%), fructose (12%), and citrate (6%). At 8 h, aspartate (60%) and fructose (27%) represented the largest modeled carbon contributions, followed by citrate (13%). Together, these predictions indicate temporal redistribution in the relative contributions of individual substrates in FEM over time, providing mechanistic insight into the observed growth phenotypes.

The top 24 reactions with the highest modeled carbon fluxes, along with substrate transport reactions, are shown in Fig. 2B. Model predictions suggest that the coordinated utilization of glucose and malate may contribute to the reduced lag observed under FEM conditions by increasing NADPH availability through malic enzyme activity during the first 3 h of growth. The mixed substrate FEM environment reduces the need for anaplerotic pathway flux to replenish TCA cycle metabolites, which would be required if *P. putida* only had access to glucose but not malate. FEM also reduces the requirement for gluconeogenesis flux that would be needed if malate was the sole carbon source. These predictions suggest that mixed substrates in FEM may facilitate rapid growth initiation by reducing metabolic constraints associated with reliance on a single carbon source.

We next used temporal proteomic data (Table S3) as an independent experimental validation of model-predicted pathway activity under FEM conditions. Proteins associated with FEM substrate transport and metabolism, including aspartate metabolism, fructose metabolism, and the TCA cycle, showed temporal abundance trends that aligned with model-predicted fluxes (Fig. 2B). These results support the ability of experimentally constrained metabolic modeling to predict pathway-level metabolic responses during growth on FEM.

KEGG pathway enrichment analysis revealed early activation of glyoxylate and dicarboxylate metabolism, along with bacterial chemotaxis, consistent with rapid metabolic adaptation to the FEM environment (Fig. S1). By 24 h, proteomics data indicated increased abundance of enzymes associated with fatty acid and amino acid degradation (Fig. S1), consistent with necromass turnover following substrate depletion. This aligns with the FEM growth kinetics (Fig. 1C) that show the cells in the death phase, and the substrate depletion profiles (Fig. 1G) that show all substrates were exhausted by this time.

Our experimental and modeling data together suggest that metabolism under FEM conditions is characterized by early substrate prioritization followed by overlapping co-utilization of multiple substrates. Early malate consumption and contribution of malate predicted by the model are consistent with studies that have shown *P. putida’s* preference for organic acids (7). Although classical catabolite repression has been reported in *P. putida* grown in complex media such as LB (16), the utilization of multiple substrates and the absence of significant changes in catabolite repression control proteins in our data suggest that catabolite repression is not the dominant regulator of metabolism under FEM conditions.

Our findings indicate that the specific composition of the FEM molecular environment shapes substrate utilization behavior and gives rise to an emergent growth phenotype. While individual substrates supported only subsets of the observed growth traits, the mixed-substrate environment combined rapid growth initiation and attainment of maximum biomass, and substantial biomass accumulation. For example, fructose alone supported the highest biomass accumulation but exhibited substantially delayed growth initiation and time to maximum biomass, whereas citrate alone reached maximum biomass on a timescale comparable to FEM but achieved significantly lower maximum cell density. In environments such as the hyphosphere, where substrates are chemically diverse and transient, microbes capable of rapidly initiating and establishing growth may gain an advantage that influences microbial competition and community assembly. Reduced lag phases are also desirable in bioprocessing applications, where rapid culture establishment can improve productivity (17).

More broadly, these findings suggest that naturally evolved molecular environments may provide useful design principles for engineering microbial growth conditions and microbial biosystems. Our results also highlight the importance of studying microbes under chemically realistic conditions, as growth phenotypes and metabolic responses observed in mixed substrate environments cannot be reliably inferred or predicted from single-substrate experiments alone.

## Supplementary Material

Reviewer comments

## Data Availability

Primary raw mass spectrometry metabolomics and proteomics data have been deposited at MassIVE under accession numbers MSV000100994 and MSV000100991 (18, 19). Proteomics expression data, including normalized mean counts and statistics for observed proteins, are provided in Table S3.

## AFTER EPUB

*[This article was published on 24 July 2026 with errors in the affiliations. The affiliations were corrected in the current version, posted on 5 August 2026.]*

## SUPPLEMENTAL MATERIAL

Supplemental Information msystems.00760-26-s0001.docx Supplemental material and methods, Tables S1 and S2, and Figure S1.

Table S3 msystems.00760-26-s0002.xlsx Proteomics data and statistics.

OPEN PEER REVIEW reviewer-comments.pdf An accounting of the reviewer comments and feedback.

ASM does not own the copyrights to Supplemental Material that may be linked to, or accessed through, an article. The authors have granted ASM a non-exclusive, world-wide license to publish the Supplemental Material files. Please contact the corresponding author directly for reuse.
