# Supplementary material for: A fungal exudate-inspired substrate mixture elicits an emergent growth phenotype and metabolic responses in Pseudomonas putida
Source: mSystems. 2026 Jul 24;11(8):e00760-26. doi: 10.1128/msystems.00760-26 (PMC13483340; doi:10.1128/msystems.00760-26)
Supplement: Supplementary file 2 [file msystems.00760-26-s0001.docx]

Supplementary Information

**TITLE**: A fungal exudate inspired substrate mixture elicits an emergent growth phenotype and metabolic responses in Pseudomonas putida

Authors: Natalie Sadler,^1,#^ Elise Van Fossen,^1,#^ Grace Black,^1^ Vanessa Paurus,^1^ Nathalie Munoz,^1^ Yuqian Gao,^1^ Isaac Kwame Attah,^1^ Damon Leach,^1^ Joonhoon Kim,^2^ Sneha Couvillion^1,*^

^1^Integrated Discovery Sciences Directorate, Pacific Northwest National Laboratory, Richland and Sequim, WA, USA

^2^Energy and Environment Directorate, Pacific Northwest National Laboratory, Richland, WA, USA

\# Co-first authors contributed equally

*Corresponding author: [sneha.couvillion@pnnl.gov](mailto:sneha.couvillion@pnnl.gov)

**MATERIALS AND METHODS**

***P. putida* Growth on Fungal Exudate Mimic (FEM) Mix**

The synthetic fungal-exudate mimic (FEM) composition was formulated based on published metabolomics profiles of fungal hyphal exudates (1, 2) from which we selected representative sugars, amino acids, and organic acids that occur at high abundances. The FEM was prepared in Modified Strullu-Roman (MSR) medium (3) with composition adjustments (described below). Glucose, fructose, trehalose, citrate, and malate were each added at 100 µg C·mL⁻^1^, while aspartic acid was included at 300 µg C·mL⁻¹. Glutamine and asparagine were initially tested but excluded from the final formulation due to unreliable GC–MS detection, likely resulting from sorption interactions with phytate. Phytate was included at 300 µM as a representative organic phosphorus source commonly found in soil (4). Additional MSR medium modifications included exchange of MnCl_2_-4H_2_O for MnSO_4_-4H_2_O and the exclusion of Ca(NO_3_)_2_-4H_2_O to prevent potential phytase inhibition (1). To approximate reported carbon-to-nitrogen ratios of fungal hyphal exudates (2), KNO₃ was increased from 0.075 mM to 11.5 mM. The resulting FEM provided a total of 800 µg C·mL⁻¹ (≈66 mM carbon) and 134 µg N·mL⁻¹ (≈9.6 mM nitrogen).

MSR media was prepared by mixing 739 mg·L^-1^ MgSO_4_,1.24 g·L^-1^ KNO_3_, 4.1 mg·L^-1^ KH_2_PO_4_, 8.0 mg·L^-1^ NaFeEDTA, 65 mg·L^-1^ KCl, 2.45 mg·L^-1^ MnCl-4H_2_O, 0.29 mg·L^-1^ ZnSO_4_-7H_2_O, 1.86 mg·L^-1^ H_3_BO_3_, 0.035 mg·L^-1^ CuSO_4_-5H_2_O, 0.0024 mg·L^-1^ (NH_4_)6Mo7O24-4H_2_O, and 198 mg·L^-1^ phytic acid sodium salt hydrate in 700 mL of sterile deionized water. Next, media pH was adjusted to 6 with 4 N HCl, and then volume was brought up to 1 L with deionized water. The media was then filter sterilized using a 0.22 µm PES membrane bottle top vacuum assisted filtration device (Fisher) and stored in the dark at room temperature. FEM was prepared by mixing 250 mg·L^-1^ glucose, 250 mg·L^-1^ fructose, 261 mg·L^-1^ D-(+)-trehalose dihydrate, 344 mg·L^-1^ sodium citrate tribasic dihydrate, 370 mg·L^-1^ sodium-L malate, and 830 mg·L^-1^ aspartic acid in MSR media, adjusted to pH 6, and filter sterilized using 0.22 µm bottle top vacuum assisted filtration device. These concentrations are 10 times higher than the approximate labile C-rich composition reported for AMF hyphal metabolite profiles (5). We also prepared a 0.1X FEM media that is in line with the reported values (5) and 0.5X FEM by diluting the FEM media with C free MSR.

*P. putida* cultures were started by streaking cells from 20% glycerol stocks stored at -80 °C on to Reasoner’s 2A (R2A; Neogen) media solidified with 1.5% agar in 100mm petri dishes and then incubated at 28 °C for 48 hours. Single colonies were transferred to 3 mL of R2A broth in 15 mL polystyrene culture tubes. Broth cultures were incubated at 28 °C with 200 rpm shaking for 24 hours. This small volume culture was used to inoculate 50 mL of R2A in a 250 mL flask which was incubated at 28 °C with 200 rpm shaking for 24 hours. Next, cells were pelleted by centrifugation (6,000 x g, 5 min) and the supernatant decanted. The cell pellets were suspended in MSR media to wash the cells and then pelleted again by centrifugation. Supernatants were decanted and C free MSR media was used to resuspend the cells and then split into three 50mL tubes. The centrifuged to pellet cells and supernatants were decanted. Cells were resuspended in either 1X, 0.5X, or 0.1X FEM in MSR and OD_600nm_ was measured by spectrophotometer and adjusted to 0.1 with corresponding FEM in MSR. 5 mL of cells for each media type were aliquoted into 15 50mL Olympus conical tubes (Genesse) (3 reps for 5 collection time points including 0, 3, 5, 8, and 24 hours). Cells for each media type were also aliquoted (200 µL of cells) in 3 wells of a clear 96 well plate. Media only controls were also included. Kinetic growth was measured by OD_600nm_ absorbance measured using a CytationC10 (Agilent) with temperature set to 28C and continuous orbital shaking at 548 cpm. The sampling time points were chosen based on previous kinetic growth curves assays prepared in this same way and run for 48 hours.

At each time point, samples were split into cell pellets and supernatants. To do so, incubation tubes were centrifuged (6,000 x g for 5 minutes,) and the supernatants were aliquoted into three 2 mL Eppendorf snap cap tubes and flash frozen in liquid N_2_. Cell pellets were flash frozen with liquid N_2_ and stored at -80C for sample preparation and LC-MS proteomics analysis.

**GC-MS analysis**

**Preparation of supernatant for metabolite quantification**

For quantification of metabolites of interest in the spent medium, 50 or 20 µl of supernatant samples were dried under speed-vacuum in a glass vial (50 µl for the 0.1 XFEM, 20 µl for all other samples). For quantification of aspartic acid, the 0.5X FEM samples were diluted by a factor of 1/2 for the 0 hr time point and the FEM supernatants were diluted by a factor of 1/3. Solutions ranging from 0 to 300 µg/mL containing standards of the sugars and organic acids of interest (glucose, fructose, citric acid, aspartic acid and malic acid) were prepared and 20 µl of these in triplicate were dried to build an external calibration curve.

**Targeted metabolomics for supernatant**

The supernatant samples and standard mix aliquots were completely dried under vacuum and chemically derivatized as previously reported (6). Briefly, the extracted metabolites were derivatized by methoxyamination and trimethylsilyation (TMS), then the samples were analyzed by GC-MS. Samples were run in an Agilent GC 7890A using a HP-5MS column (30 m × 0.25 mm × 0.25 μm; Agilent Technologies, Santa Clara, CA) coupled with a single quadrupole MSD 5975C (Agilent Technologies). One microliter of sample was injected into a splitless port at constant temperature of 250°C. The GC temperature gradient started at 60 °C and held for 1 minute after injection, followed by increase to 325 °C at a rate of 10 °C/minute and a 5-minute hold at this temperature. A fatty acid methyl ester mix (C8-28) (Sigma-Aldrich) was analyzed in parallel as standard for retention time calibration. GC-MS raw data files were processed using the Metabolite Detector software (7). Retention indices (RI) of detected metabolites were calculated based on the analysis of a FAMEs mixture, followed by their chromatographic alignment across all analyses after deconvolution. Metabolites were identified by matching experimental spectra to a PNNL augmented version of Agilent GC-MS metabolomics Library. Calibration curves were established for each of the standards in the standard mixture, based on peak areas of ions characteristic of those metabolites. After linear regression fitting, concentration of the metabolites in the spent medium samples was determined upon adjustment to the dilution factors and dried volumes.

**Metabolic modeling**

The genome-scale metabolic model of *P. putida* iJN1462(8) was used for the dynamic flux balance analysis of *P. putida* metabolism in the FEM environment. The iJN1462 model was augmented with a periplasmic phytase reaction and transport reactions for phytate and myo-inositol enabling explicit representation of phytate hydrolysis in the periplasm and subsequent uptake of soluble inositol phosphates, thereby extending the model to account for phosphorus acquisition from complex organic sources. The uptake of FEM substrates was modeled using Michaelis-Menten kinetics where the specific uptake rate, *q_S_*, for each substrate *S* is determined from the maximum rate (*V_max_*), Michaelis constant (*K_M_*), and substrate concentration [*S*].

*q_S_* = *V_max_* [*S*] / (*K_M_* + [*S*])

To estimate the kinetic parameters *V_max_*, and *K_M_*, least squares optimization was employed to minimize the sum of squares of the differences between model predicted and experimentally observed concentration profiles of substrates and cells over the 24-hour time-course with three different initial FEM concentrations. The 24-hour time-course was divided into 24 intervals, and a flux balance analysis (FBA) problem was solved for each interval assuming that the metabolic fluxes are constant over the interval. The Initial concentrations of fungal exudate components, phytate, and cells were used for calculating uptake rates using Michaelis-Menten kinetics and simulating cell growth using FBA during the first time interval. For subsequent time intervals, the concentrations of substrates and cells were updated assuming exponential cell growth and the process were repeated.

[*X*]*_t+Δt_* = [*X*]*_t_* e*^µΔt^*

[*S*]*_t+Δt_* = [*S*]*_t_* - *q_S_* /*m* *(e*^µΔt^* - 1)

The estimated kinetic parameters were used to constrain substrate uptake and predict intracellular flux by solving parsimonious FBA (https://doi.org/10.1038/msb.2010.47) for each time interval. The least squares optimization was performed using the LMFIT package in python (9) and the FBA problems were solved using the COBRApy package(10) in python.

**Proteomics Analysis**

**Chemicals**: Chemicals were obtained from Sigma-Aldrich (St. Louis, MO) with the following exceptions: Dithiothreitol, bicinchoninic acid protein assay (BCA), and iodoacetamide were obtained from ThermoFisher Scientific (Waltham, MA). Trypsin was obtained from USB (formerly Affymetryx, now ThermoFisher Scientific). Water used was ultrapurified water obtained from a Millipore Milli-Q system (St. Louis, MO).

**Sample extractions**: A chloroform:methanol extraction (Nakayasu et. al. PMCID: PMC5069757) was performed on lyophilized supernatants and *P. putida* cell pellets. Briefly, 200µL of ice-cold ultrapure water was added to each sample in a 1.7-mL Sorenson tube (Sorenson Bioscience Inc, Murray, UT). Following water, 1 mL of ice-cold chloroform was added to each sample and the tubes were vortexed for 1 minute. All samples were placed on ice for 5 minutes followed by another 1-minute vortex. The samples were then centrifuged at 10K x g for 10 min at 4°C. The top (polar) layer was transferred to a new 1.7mL Sorenson tube, while the lower (non-polar) layer was transferred to an ALS vial (Waters Corp., Milford, MA). Both the polar and non-polar layers were stored at -80°C. The precipitated protein interphase was washed with 1.0-mL of ice-cold methanol, and centrifuged at 15,000 x g, 4°C for 10 minutes. The methanol was decanted, and the pellet allowed to dry by inverting the tube over a kimwipe for ~5 minutes. Denaturing buffer (8M urea in 50 mM ammonium bicarbonate) was added to each protein pellet. The pellets were reconstituted to a soluble homogenate using vortexing and brief sonication, then a BCA protein assay was performed in a 96 well plate to determine approximate protein concentration of each sample by measuring absorbance at 562_nm_ on a plate reader. Dithiothreitol was added to a 5 mM concentration and the samples were incubated on a Thermomixer (Eppendorf) for 30 minutes at 60°C with shaking at 500 rpm. Iodoacetamide was added to a 40 mM concentration and the samples were incubated for 30 minutes at 25°C with shaking at 500rpm in the dark. Samples were diluted eight-fold with 1.14 mM CaCl_2_ in 50 mM ammonium bicarbonate and trypsin was added in a 1:50 (trypsin:protein, w:w) ratio. The samples were incubated overnight at 37°C with shaking at 500 rpm, then frozen and stored at -80°C. Solid Phase Extraction (SPE) was performed on the samples using Waters Sep-pak 40mg plate. Sample was acidified to 0.1% with TFA (Trifluoroacetic acid). Columns were conditioned twice with 600µL of methanol followed by 600uL of 50% Acetonitrile (ACN), 0.1% FA (Formic Acid). Columns were washed three times with 0.1% TFA in water. Samples were applied to each column followed by 600 µL of 0.1% TFA three times. Samples were washed one more time with 600 µL of 1% FA and then eluted into 1.5-mL microcentrifuge tubes with 50% ACN, 0.1% FA and concentrated in a vacuum concentrator to 50 µL. Another BCA protein assay was performed to obtain peptide concentration and a portion of each sample was diluted to 0.1 µg/µL for LC-MS analysis.

**Instrument analysis**: Sample peptides were analyzed using the Orbitrap Astral mass spectrometer (Thermo Scientific), with full scan spectra acquired using the Orbitrap analyzer over a scan range of 380 to 980 m/z, at 240000 resolution. The normalized AGC target was set at 500%, maximum injection time set at 5 ms, and the RF lens set at 45%. For the nDIA settings, the DIA window type was set to “Auto”, window placement optimization set to “On”, window overlap at 0, and the isolation window at 2 m/z. Higher-energy collisional dissociation was performed at a normalized collision energy of 25% with loop control set at 0.6 seconds.

Liquid chromatography separation was performed using a Vanquish Neo LC (Thermo Scientific), with the LC interfaced to the Astral MS using the EASY-Spray ion source (Thermo Scientific), the spray voltage set at 2.2 kV, and the Ion transfer tube temperature set to 300°C. The LC was run using a 70 SPD (Samples Per Day) separation method, with each sample analyzed using a 14-minute active gradient. The Vanquish Neo was operated in the trap-and-elute mode, using a PepMap ES906 analytical column for reverse phase elution and the PepMap Neo Trap Cartridge (Thermo Scientific) for sample trapping operated in the “backwards flush” mode. Mobile phases consisted of (A) 0.1% formic acid in water and (B) 0.1% formic acid in acetonitrile with the following gradient profile (min, Flow Rate, %B): 0.0, 3.50,1.0; 0 .001, 2.80, 1.0; 0 .501, 1.30, 4.0; 0 .901, 1.10, 8.0; 12.301, 1.10, 22.5; 15.301, 1.10, 35.0; 15.501, 1.10, 55.0; Column Wash; 16.001, 2.80, 99.0; 17.001, 2.80, 99.0; 18.000, 2.80, 1.0.

**Data analysis and statistics**: Proteomics data was performed using DIA-NN (version 2.2.0 Academia). The reference *Pseudomonas putida* KT2440 proteome (UniProt UP000000556, release 2025_01) was used for in silico digestion (fully tryptic peptides, 7-30 amino acids, up to two missed cleavages), precursor m/z range 380-930, and charge states 2-4. Static carbamidomethylation (+57.9949 Da) was applied to cysteine, while dynamic modifications included protein N-terminal acetylation (+42.0106 Da) and methionine oxidation (+15.9949 Da), with up to three variable modifications per peptide. DIA-NN generated an empirical spectral library and performed match-between-runs (MBR) with heuristic protein grouping. Identifications were filtered at 1% FDR for precursors and protein groups at both run-specific (Q.Value, PG.Q.Value) and library entry levels (Lib.Q.Value, Lib.PG.Value), with additional quality thresholds (Quatity.Quanlity ≥ 0.5, PG.MaxLFQ.Quality ≥ 0.7) to ensure robust quantification.

Proteomics data were initially processed at the peptide level. Missing values were encoded as NA and data were log2 transformed. Peptides with a single observation across all samples were removed. A robust Mahalanobis distance was calculated for each sample to identify potential sample outliers using the metrics skewness of the sample, median absolute deviance (MAD) of the sample, and the proportion of missing values in the sample. Data were normalized via global median centering. Peptide data were then rolled up to the protein level using median reference rollup. Data were assessed via ANOVA and G-test to identify differences between time points.

**SUPPLEMENTARY TABLES**

**Supplementary Table S1**. Temporal GC-MS profiling of spent FEM medium to quantify substrate uptake dynamics over time

|  | **Metabolite concentration in µg/ml** | | | |  |
| --- | --- | --- | --- | --- | --- |
| **Sample** | **glucose** | **fructose** | **aspartic acid** | **citric acid** | **malic acid** |
| PPICv_0pt1XFEM_300P_00hr_A_16 | 5.5 | 6.8 | 10.8 | 1.7 | 4.6 |
| PPICv_0pt1XFEM_300P_00hr_B_17 | 2.7 | 4.1 | 5.4 | 1.3 | 3.1 |
| PPICv_0pt1XFEM_300P_00hr_C_18 | 8.6 | 10.1 | 8.1 | 2.1 | 5.5 |
| PPICv_0pt1XFEM_300P_03hr_A_34 | 5.3 | 9.1 | 3.1 | 0.8 | 0.9 |
| PPICv_0pt1XFEM_300P_03hr_B_35 | 5.0 | 10.1 | 3.6 | 1.4 | 0.9 |
| PPICv_0pt1XFEM_300P_03hr_C_36 | 6.8 | 11.8 | 3.3 | 1.1 | 0.9 |
| PPICv_0pt1XFEM_300P_05hr_A_52 | 0.0 | 0.8 | 2.8 | 0.6 | 0.9 |
| PPICv_0pt1XFEM_300P_05hr_B_53 | 0.0 | 0.9 | 2.8 | 0.6 | 0.9 |
| PPICv_0pt1XFEM_300P_05hr_C_54 | 0.0 | 0.8 | 2.8 | 0.6 | 0.9 |
| PPICv_0pt1XFEM_300P_08hr_A_70 | 0.0 | 0.6 | 2.8 | 0.6 | 0.9 |
| PPICv_0pt1XFEM_300P_08hr_B_71 | 0.0 | 0.6 | 2.8 | 0.6 | 0.9 |
| PPICv_0pt1XFEM_300P_08hr_C_72 | 0.0 | 0.6 | 2.8 | 0.6 | 0.9 |
| PPICv_0pt1XFEM_300P_24hr_A_88 | 0.0 | 0.6 | 2.9 | 0.6 | 0.9 |
| PPICv_0pt1XFEM_300P_24hr_B_89 | 0.0 | 0.6 | 2.9 | 0.6 | 0.9 |
| PPICv_0pt1XFEM_300P_24hr_C_90 | 0.0 | 0.6 | 2.9 | 0.6 | 0.9 |
| PPICv_0pt5XFEM_300P_00hr_A_10 | 120.1 | 142.3 | 422.5 | 79.4 | 142.2 |
| PPICv_0pt5XFEM_300P_00hr_B_11 | 112.2 | 128.9 | 298.2 | 70.5 | 127.7 |
| PPICv_0pt5XFEM_300P_00hr_C_12 | 108.0 | 122.2 | 243.2 | 63.5 | 114.4 |
| PPICv_0pt5XFEM_300P_03hr_A_28 | 116.5 | 148.1 | 374.8 | 109.0 | 23.3 |
| PPICv_0pt5XFEM_300P_03hr_B_29 | 101.9 | 127.5 | 350.8 | 89.2 | 22.7 |
| PPICv_0pt5XFEM_300P_03hr_C_30 | 97.1 | 123.2 | 353.6 | 109.6 | 25.9 |
| PPICv_0pt5XFEM_300P_05hr_A_46 | 93.9 | 130.2 | 304.7 | 61.3 | 3.2 |
| PPICv_0pt5XFEM_300P_05hr_B_47 | 87.9 | 120.5 | 277.8 | 58.2 | 3.0 |
| PPICv_0pt5XFEM_300P_05hr_C_48 | 78.3 | 111.0 | 257.3 | 47.0 | 2.9 |
| PPICv_0pt5XFEM_300P_08hr_A_64 | 4.5 | 38.0 | 36.5 | 13.4 | 2.4 |
| PPICv_0pt5XFEM_300P_08hr_B_65 | 3.9 | 36.1 | 25.1 | 7.8 | 2.2 |
| PPICv_0pt5XFEM_300P_08hr_C_66 | 5.7 | 41.2 | 36.4 | 9.8 | 2.2 |
| PPICv_0pt5XFEM_300P_24hr_A_82 | 0.0 | 1.5 | 7.1 | 1.5 | 2.2 |
| PPICv_0pt5XFEM_300P_24hr_B_83 | 0.0 | 1.5 | 7.1 | 1.5 | 2.2 |
| PPICv_0pt5XFEM_300P_24hr_C_84 | 0.0 | 1.5 | 7.2 | 1.5 | 2.2 |
| PPICv_1XFEM_300P_00hr_A_94 | 238.1 | 224.1 | 923.8 | 199.3 | 310.4 |
| PPICv_1XFEM_300P_00hr_B_95 | 221.9 | 210.6 | 793.7 | 179.8 | 283.5 |
| PPICv_1XFEM_300P_00hr_C_96 | 177.9 | 164.7 | 678.6 | 130.0 | 211.4 |
| PPICv_1XFEM_300P_03hr_A_22 | 175.8 | 250.2 | 746.5 | 213.0 | 204.6 |
| PPICv_1XFEM_300P_03hr_B_23 | 172.8 | 244.3 | 818.7 | 191.9 | 189.5 |
| PPICv_1XFEM_300P_03hr_C_24 | 181.6 | 261.6 | 810.8 | 223.0 | 216.2 |
| PPICv_1XFEM_300P_05hr_A_40 | 168.6 | 238.9 | 699.4 | 196.6 | 62.9 |
| PPICv_1XFEM_300P_05hr_B_41 | 172.3 | 249.1 | 762.8 | 201.9 | 67.9 |
| PPICv_1XFEM_300P_05hr_C_42 | 181.0 | 261.3 | 771.2 | 210.3 | 80.5 |
| PPICv_1XFEM_300P_08hr_A_58 | 62.4 | 189.4 | 653.3 | 138.0 | 3.4 |
| PPICv_1XFEM_300P_08hr_B_59 | 83.7 | 229.6 | 773.5 | 167.8 | 3.0 |
| PPICv_1XFEM_300P_08hr_C_60 | 84.4 | 249.8 | 624.9 | 177.2 | 3.2 |
| PPICv_1XFEM_300P_24hr_A_76 | 0.0 | 1.5 | 318.6 | 1.5 | 2.4 |
| PPICv_1XFEM_300P_24hr_B_77 | 0.0 | 1.5 | 316.4 | 1.5 | 2.3 |
| PPICv_1XFEM_300P_24hr_C_78 | 0.0 | 1.6 | 336.0 | 1.5 | 2.4 |

**Supplementary Table S2**. Estimated Michaelis-Menten parameter values for FEM substrates and their standard errors

|  | Vmax (mmol/gDW/hr) | Km (mM) |
| --- | --- | --- |
| Glucose | 0.692 ± 0.240 | 0.0018 ± 0.1519 |
| Fructose | 0.172 ± 0.122 | 0.0482 ± 0.2865 |
| Aspartic acid | 0.548 ± 0.177 | 0.0337 ± 1.0553 |
| Citric acid | 0.090 ± 0.164 | 0.0243 ± 0.3742 |
| Malic acid | 2.482 ± 0.928 | 0.0028 ± 0.2608 |

**SUPPLEMENTARY FIGURES**

**Supplementary Figure S1**. KEGG enrichment of differentially expressed proteins in 5, 8, and 24 hours compared to 3 hours.

**REFERENCES**

1.	Zhang L, Feng G, Declerck S. 2018. Signal beyond nutrient, fructose, exuded by an arbuscular mycorrhizal fungus triggers phytate mineralization by a phosphate solubilizing bacterium. The ISME Journal 12:2339-2351.

2.	Li X, Zhao R, Li D, Wang G, Bei S, Ju X, An R, Li L, Kuyper TW, Christie P, Bender FS, Veen C, van der Heijden MGA, van der Putten WH, Zhang F, Butterbach-Bahl K, Zhang J. 2023. Mycorrhiza-mediated recruitment of complete denitrifying Pseudomonas reduces N2O emissions from soil. Microbiome 11:45.

3.	Declerck S, Strullu DG, Plenchette C. 1998. Monoxenic culture of the intraradical forms of Glomus sp. isolated from a tropical ecosystem: a proposed methodology for germplasm collection. Mycologia 90:579-585.

4.	Jiang F, Zhang L, Zhou J, George TS, Feng G. 2021. Arbuscular mycorrhizal fungi enhance mineralisation of organic phosphorus by carrying bacteria along their extraradical hyphae. New Phytologist 230:304-315.

5.	Anonymous. !!! INVALID CITATION !!! (2).

6.	Couvillion SP, Danczak RE, Cao X, Yang Q, Keerthisinghe TP, McClure RS, Bitounis D, Burnet MC, Fansler SJ, Richardson RE, Fang M, Qian W-J, Demokritou P, Thrall BD. 2023. Graphene oxide exposure alters gut microbial community composition and metabolism in an in vitro human model. NanoImpact 30:100463.

7.	Hiller K, Hangebrauk J, Jäger C, Spura J, Schreiber K, Schomburg D. 2009. MetaboliteDetector: Comprehensive Analysis Tool for Targeted and Nontargeted GC/MS Based Metabolome Analysis. Analytical Chemistry 81:3429-3439.

8.	Nogales J, Mueller J, Gudmundsson S, Canalejo FJ, Duque E, Monk J, Feist AM, Ramos JL, Niu W, Palsson BO. 2020. High-quality genome-scale metabolic modelling of Pseudomonas putida highlights its broad metabolic capabilities. Environmental Microbiology 22:255-269.

9.	Newville M, Otten, R., Nelson, A., Stensitzki, T., Ingargiola, A., Allan, D., Fox, A., Carter, F., & Rawlik, M. . 2025. LMFIT: Non-Linear Least-Squares Minimization and Curve-Fitting for Python (1.3.4). Zenodo. <https://doi.org/10.5281/zenodo.16175987>.

10.	Ebrahim A, Lerman JA, Palsson BO, Hyduke DR. 2013. COBRApy: COnstraints-Based Reconstruction and Analysis for Python. BMC Systems Biology 7:74.
